# Frontal Headache – An Unusual Presentation of Pneumomediastinum

**DOI:** 10.5811/cpcem.2017.7.34497

**Published:** 2017-10-03

**Authors:** Robert Root, Bryant M. Bullock, Kevin Schlicksup

**Affiliations:** * William Beaumont Army Medical Center, Department of Emergency Medicine, El Paso, Texas; † Madigan Army Medical Center, Fort Lewis, Washington; ‡ Carl R. Darnall Army Medical Center, Department of Emergency Medicine, Fort Hood, Texas

## CASE PRESENTATION

**Image 1:**
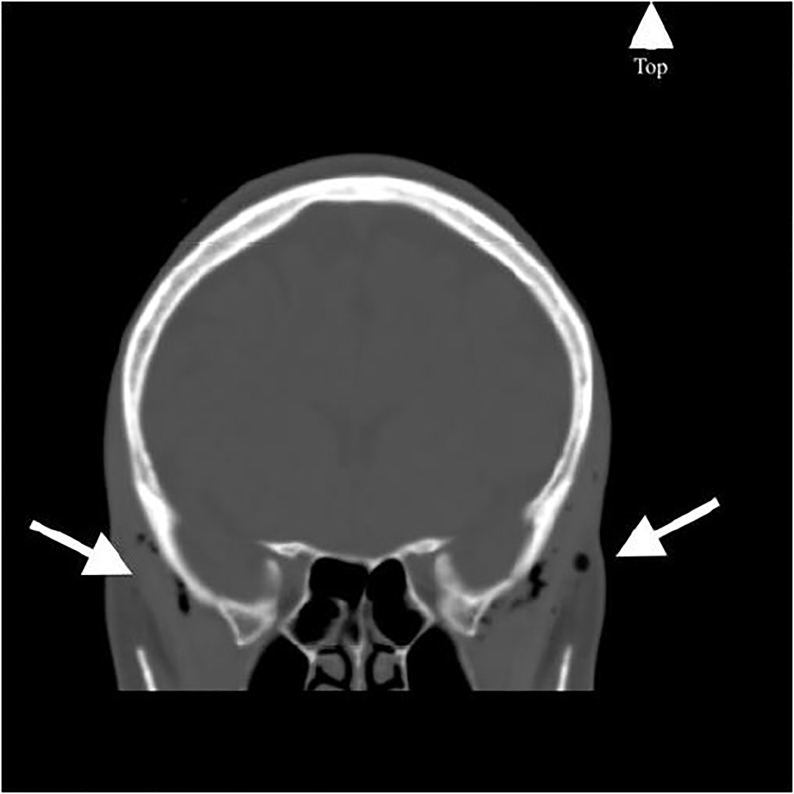
Coronal computed tomography of the brain without contrast demonstrating bilateral air tracking into the subcutaneous temporal tissue (arrows).

**Image 2:**
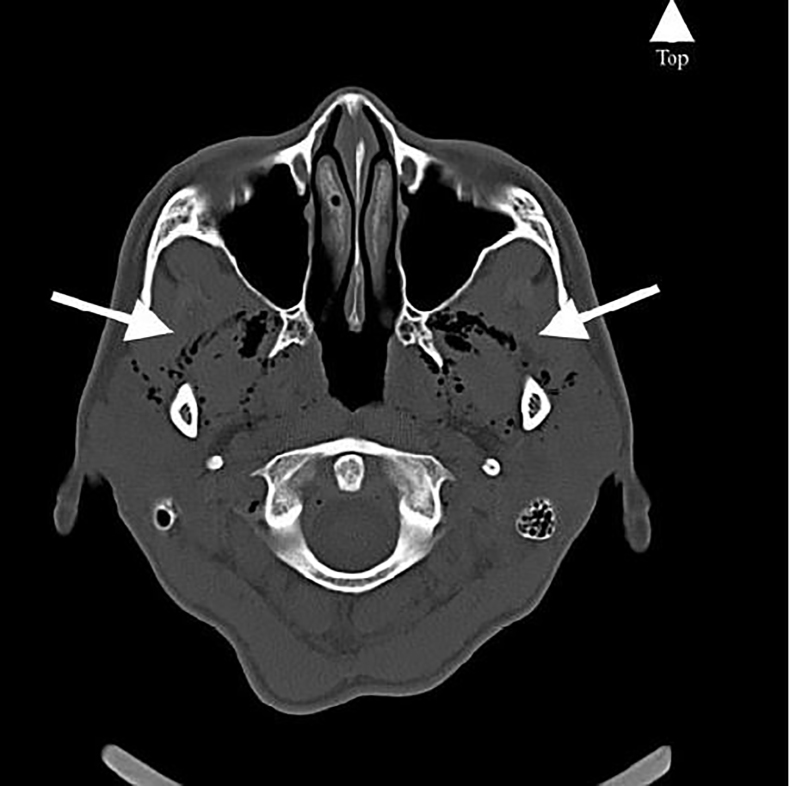
Axial computed tomography of the brain without contrast demonstrating bilateral air tracking into the pterygoid muscles (arrows).

A 45-year-old woman with past medical history of asthma presented to the emergency department with four days of pulsatile, frontal headache, different in character and intensity from her usual tension-type headaches. She reported the onset of pain as gradual without an inciting event. Her vital signs were within normal limits. She had no meningeal signs or neurologic deficits. Computed tomography (CT) of the brain without contrast demonstrated bilateral air tracking in the subcutaneous temporal tissue (Image 1) and along the pterygoid muscles (Image 2). Subsequent CT of the chest and neck revealed a small pneumomediastinum tracking upward into the head. On re-examination, no palpable subcutaneous emphysema was appreciated after detailed search in the chest, neck, and head. She denied trauma, illicit drug use, being at altitude, diving, or recent surgery or intubation. Apart from headache, she remained asymptomatic without chest pain or dyspnea. She was admitted to the hospital for 24-hour observation. Follow-up CT five days later demonstrated resolution of all findings, with improvement in the patient’s headache. Her uneventful observation ruled out life-threatening etiologies (mediastinitis, tracheoesophageal injury) and pneumomediastinum was ultimately attributed to increased intrathoracic pressure secondary to her poorly controlled asthma.

## DISCUSSION

Spontaneous pneumomediastinum is the presence of gas in the mediastinum in the absence of trauma.^1^ Although rare, it’s a recognized complication of poorly controlled asthma, inhalation of illicit drugs, vigorous vomiting or coughing, strenuous exercise, and Valsalva maneuver.^1^ It occurs when increased intrathoracic pressure results in alveolar rupture, allowing for dissection of air into the mediastinum.^2^ Patients typically present with chest pain or dyspnea.^1^,^2^,^3^ The most common findings include subcutaneous emphysema, Hamman’s sign, and dyspnea.^1^ The condition typically self resolves and rarely has severe complications.^3^,^4^ Apart from our patient’s history of asthma, she had none of the typical findings. This case succinctly demonstrates how patients ignore our textbooks when they come to us seeking care, and that common chief complaints can hide uncommon pathology.

**CPC-EM Capsule**

What do we already know about this clinical entity?

The authors are unaware of another case where spontaneous pneumomediastinum secondary to poorly controlled asthma presented with the chief complaint of headache.

What is the major impact of the image(s)?

These images provide a unique underlying cause to a common chief complaint encountered by the emergency physician.

How might this improve emergency medicine practice?

The case is a reminder that patients continue to ignore textbooks when they seek care, and that common chief complaints can hide uncommon pathology.
